# Evaluating the Role of Microbial Internal Storage Turnover on Nitrous Oxide Accumulation During Denitrification

**DOI:** 10.1038/srep15138

**Published:** 2015-10-14

**Authors:** Yiwen Liu, Lai Peng, Jianhua Guo, Xueming Chen, Zhiguo Yuan, Bing-Jie Ni

**Affiliations:** 1 Advanced Water Management Centre, The University of Queensland, St. Lucia, Brisbane, QLD 4072, Australia

## Abstract

Biological wastewater treatment processes under a dynamic regime with respect to carbon substrate can result in microbial storage of internal polymers (e.g., polyhydroxybutyrate (PHB)) and their subsequent utilizations. These storage turnovers play important roles in nitrous oxide (N_2_O) accumulation during heterotrophic denitrification in biological wastewater treatment. In this work, a mathematical model is developed to evaluate the key role of PHB storage turnovers on N_2_O accumulation during denitrification for the first time, aiming to establish the key relationship between N_2_O accumulation and PHB storage production. The model is successfully calibrated and validated using N_2_O data from two independent experimental systems with PHB storage turnovers. The model satisfactorily describes nitrogen reductions, PHB storage/utilization, and N_2_O accumulation from both systems. The results reveal a linear relationship between N_2_O accumulation and PHB production, suggesting a substantial effect of PHB storage on N_2_O accumulation during denitrification. Application of the model to simulate long-term operations of a denitrifying sequencing batch reactor and a denitrifying continuous system indicates the feeding pattern and sludge retention time would alter PHB turnovers and thus affect N_2_O accumulation. Increasing PHB utilization could substantially raise N_2_O accumulation due to the relatively low N_2_O reduction rate when using PHB as carbon source.

Heterotrophic denitrification is an important process in biological nitrogen removal, which is the widely practiced approach for nitrogen removal from wastewater. The complete denitrification includes sequential reductions of nitrate (NO_3_^−^) to dinitrogen gas (N_2_) with nitrite (NO_2_^−^), nitric oxide (NO) and nitrous oxide (N_2_O) as intermediates, carried out by heterotrophic denitrifiers. Each reduction step is catalyzed by the corresponding denitrification reductase, namely nitrate reductase (Nar), nitrite reductase (Nir), NO reductase (Nor) and N_2_O reductase (Nos), respectively^1^. As an intermediate, N_2_O can accumulate during denitrification under certain conditions, which has raised increasing concerns owing to its potent greenhouse gas effect and its ability to deplete stratospheric ozone^2^,^3^. The accumulated N_2_O in denitrification during anoxic phase would be stripped out during subsequent aeration, contributing to significant N_2_O emission from wastewater treatment. The accumulated N_2_O in denitrification during anoxic phase would be stripped out during subsequent aeration, significantly contributing to N_2_O emission from wastewater treatment and the carbon footprint of wastewater treatment plants (WWTP). It should be noted that an emission factor of 1% would increase the carbon footprint of a WWTP by about 30%^3^.

Biological wastewater treatment processes are usually experiencing dynamic conditions, with the microorganisms regularly experiencing rapid change of the availability of nutrients (feast/famine regime with respect to the carbon substrate)^4^,^5^, such as sequencing batch reactor (SBR) and enhanced biological phosphorus removal (EBPR) processes. Hence, it is usual for the denitrifying microorganisms to encounter feast and famine regimes during denitrification, i.e., the conditions with and without exogenous carbon substrate available. It has been known that denitrifiers respond to these dynamic conditions in wastewater treatment by forming internal storage polymers (e.g. polyhydroxybutyrate (PHB))^6^. During the feast period where the available carbon substrates largely exceed the requirement of microbial assimilatory process, the heterotrophic denitrifiers are able to simultaneously convert carbon substrate to internal storage polymer during their growth. In the following famine period where external carbon substrate is not available, the produced storage polymer can be utilized for secondary biomass growth^7^,^8^,^9^.

The production and consumption of PHB have been found to have a significant influence on N_2_O accumulation during denitrification. Schalk-Otte *et al.*^10^. reported the increase of N_2_O production from denitrification coincided with the onset of storage compound usage upon COD depletion. In this system, up to 32–64% nitrogen was emitted as N_2_O when the bacteria started to consume internal storage compounds. A highest N_2_O emission was observed in a denitrifying culture with PHB as the electron donor in comparison to the usage of other electron donors (e.g. methanol and acetate)^11^. Scherson *et al.*^12^ also managed to fulfill partial heterotrophic reduction of NO_2_^−^ to N_2_O by using PHB as the electron donor in a denitrifying lab-scale reactor. These results indicated PHB storage might play an essential role in N_2_O accumulation during denitrification. However, there has been a lack of understanding on the dependency of N_2_O accumulation on the microbial internal storage turnovers during denitrification, and the mechanisms behind this phenomenon are still unclear and need to be further clarified.

Mathematical modeling is of great significance towards understanding metabolic mechanism of N_2_O accumulation during denitrification as well as estimating site-specific N_2_O production in wastewater treatment systems. So far, several denitrification models have been proposed to describe N_2_O accumulation during denitrification^13^,^14^,^15^. For example, the Activated Sludge Model for Nitrogen (ASMN) by Hiatt *et al.*^14^ presented a typical four-step denitrification model including sequential reductions from NO_3_^−^ to N_2_ via NO_2_^−^, NO and N_2_O. In contrast to the existing denitrification models that directly couple carbon oxidation and nitrogen reduction, another denitrification model concept proposed by Pan *et al.*^15^ described these two types of processes separately through introducing electron carriers to link them for each step of denitrification. However, none of these currently available denitrifying models for N_2_O accumulation specifically considered the potential effects of anoxic PHB storage turnovers on N_2_O accumulation.

This study aims to develop a new denitrification model for describing N_2_O accumulation with the consideration of effects of microbial internal storage turnovers and evaluate the key role of storage production and utilization on N_2_O accumulation during denitrification. The model is calibrated and validated using experimental data from two different denitrifying cultures. The model is also applied to simulate a denitrifying sequencing batch reactor (SBR) and a denitrifying continuous system to reveal the impacts of the feeding pattern and sludge retention time (SRT) on storage formation as well as N_2_O accumulation.

## Results

### Model calibration

**Figure 1:**
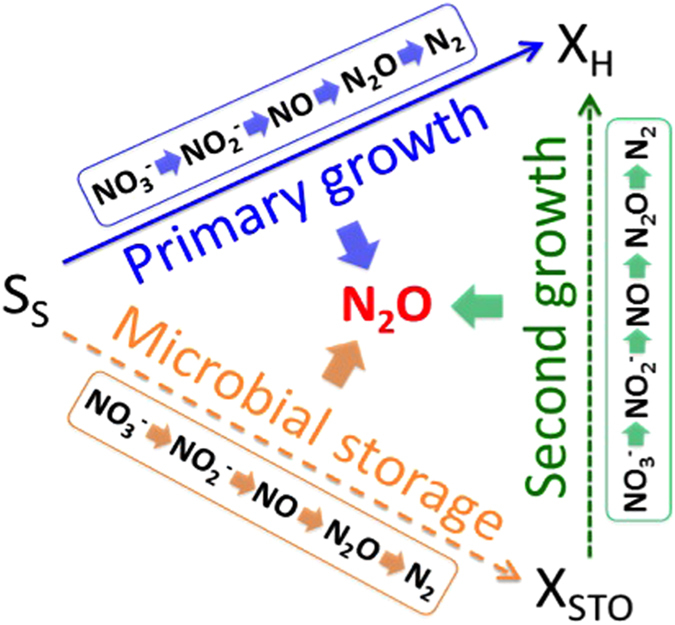
Schematic representation of the proposed four-step denitrifying N_2_O model concept with the consideration of microbial internal storage turnovers.

**Figure 2:**
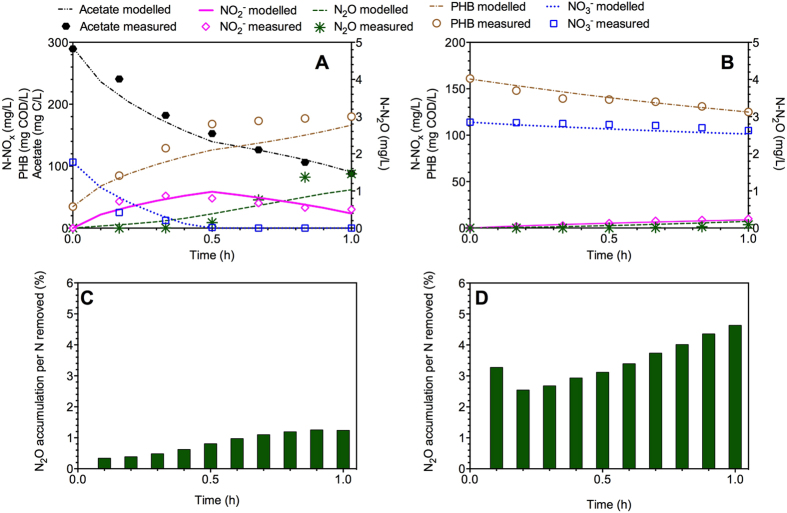
Model calibration results using the experimental data from *Denitrifying Culture I*. (**A**) NO_3_^−^, NO_2_^−^, N_2_O, acetate and PHB dynamics in Batch I (feast); (**B**) NO_3_^−^, NO_2_^−^, N_2_O and PHB profiles in Batch II (famine); (**C**) N_2_O accumulation per nitrogen removed in Batch I; and (**D**) N_2_O accumulation per nitrogen removed in Batch II.

The model developed in this work considered the four-step (from NO_3_^−^ to N_2_ via NO_2_^−^, NO and N_2_O) simultaneous anoxic storage and growth processes as well as four-step anoxic storage utilization processes for describing all potential N_2_O accumulation steps in denitrification (Fig. 1 and Table S1 and S2 in Supporting Information (SI)). The calibration of the new model involved optimizing key parameter values by fitting simulation results to the experimental data from batch tests I and II (Table 1) under both feast and famine conditions of *Denitrifying Culture I*^11^. The predicted nitrate, nitrite, PHB and N_2_O profiles with the established model are illustrated in Fig. 2, together with the experimental results. Under the feast condition (Fig. 2A), N_2_O increased gradually at the beginning, along with the accumulation of nitrite from nitrate reduction. Afterwards, N_2_O did not show significant change, consistent with the relatively stable nitrite concentrations in the remaining period. Simultaneously, PHB was produced from acetate and showed a similar trend as that of N_2_O. In comparison, under the famine condition without external carbon source (Fig. 2B), PHB served as sole electron donors for denitrification process, resulting in the accumulation of both nitrite and N_2_O continuously. Our model captured all these trends reasonably well. The good agreement between these simulated and measured data supported that the developed model properly captures the relationships among N_2_O dynamics, nitrogen reduction, and PHB turnovers for *Denitrifying Culture I*.

The calibrated parameter values giving the optimum model fittings with the experimental data are listed in Table S3 in SI. The calibrated *μ*_*1,HB,SS*_ value is higher than *μ*_*2,HB,SS*_ and *μ*_*4,HB,SS*_ values, indicating a higher nitrate reduction rate compared to nitrite and N_2_O reduction rates for this culture. The obtained value of *μ*_*storage*_ is 0.03 h^−1^, which is comparable to the literature reported value (0.02 h^−1^)^16^. The estimated *μ*_*1,HB,Xsto*_, *μ*_*2,HB,Xsto*_ and *μ*_*4,HB,Xsto*_ values are 0.033 h^−1^, 0.024 h^−1^ and 0.023 h^−1^, respectively, in agreement with Ni *et al.*^16^ who considered the anoxic growth on PHB as a two-step denitrification with a rate of 0.02 h^−1^. The lower anoxic growth rate on PHB with N_2_O as electron acceptor (*μ*_*4,HB,Xsto*_) than with others as electron acceptor (*μ*_*1,HB,Xsto*_, *μ*_*2,HB,Xsto*_ and *μ*_*3,HB,Xsto*_) led to much higher N_2_O accumulation as per nitrogen removed (2.5–5.5%, Fig. 2D) under the famine condition than that under the feast condition (0.4–1.3%, Fig. 2C) with higher N_2_O reduction rate using external substrate.

### Model validation and further evaluation

Model and parameter validation was performed based on the comparison between the model predictions (using the same parameters shown in Table S3 in SI) and the experimental data from both Batch tests III (feast condition) and IV (famine condition) (Table 1) in the presence of initial nitrite (different initial conditions) of *Denitrifying Culture I* (not used for model calibration).

**Figure 3:**
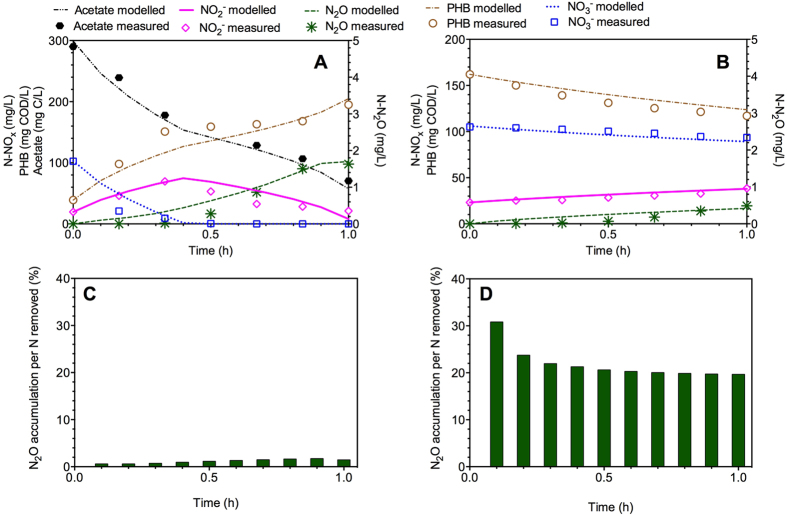
Model validation results using the experimental data from *Denitrifying Culture I* in the presence of initial nitrite. (**A**) NO_3_^−^, NO_2_^−^, N_2_O, acetate and PHB dynamics in Batch III (feast); (B) NO_3_^−^, NO_2_^−^, N_2_O and PHB profiles in Batch IV (famine); (**C**) N_2_O accumulation per nitrogen removed in Batch III; and (**D**) N_2_O accumulation per nitrogen removed in Batch IV.

The model and its parameters were first evaluated with the NO_3_^−^, NO_2_^−^, N_2_O and PHB data from Batch III (feast). The model predictions and the experimental results are shown in Fig. 3A. The validation results show that the model predictions mostly match the measured data in the validation experiment, which supports the validity of the developed model. The difference of N_2_O profiles between model predictions and the experimental results during the initial 0.5 h might be attributed to possible unrevealed N_2_O mechanisms in the system that are not considered in the model or potential experimental measurement issues, as N_2_O should be immediately accumulated with the presence of nitrite initially according to current known N_2_O mechanisms that was implemented in the model.

The developed model and the parameters were then evaluated with the NO_3_^−^, NO_2_^−^, N_2_O and PHB data from Batch IV (famine). The model predictions well matched the experimental results during the famine stage in the presence of initial nitrite as shown in Fig. 3B, again supporting the validity of the developed model. In addition, a much higher N_2_O accumulation as per nitrogen removed (20–30%) during the famine stage can be observed in Fig. 3D, in contrast to 0.5–1.5% during the feast regime (Fig. 3C), further suggesting that N_2_O accumulation during denitrification could be substantially enhanced by PHB utilization with a low N_2_O reduction rate under the famine condition.

**Figure 4:**
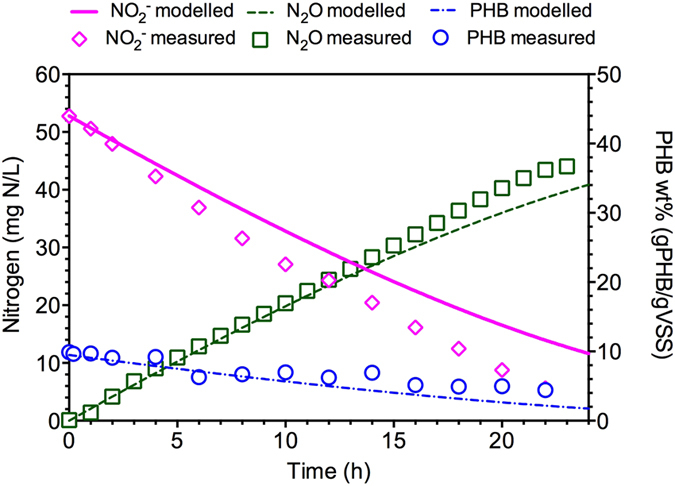
Model evaluation results using the typical cycle experimental profiles from *Denitrifying Culture II*: nitrite reduction, PHB utilization and N_2_O accumulation.

The experimental results obtained from *Denitrifying Culture II* were also used to evaluate the developed model with nitrite as only nitrogen source during the famine stage in terms of NO_2_^−^, N_2_O and PHB dynamics^17^. Two key parameters (*μ*_*2,HB,Xsto*_ and *μ*_*4,HB,Xsto*_) values were calibrated for this culture. The obtained *μ*_*2,HB,Xsto*_ value is 0.05 h^−1^, comparable with that of 0.024 h^−1^ for *Denitrifying Culture I*. The calibrated *μ*_*4,HB,Xsto*_ is 0.0004 h^−1^, nearly two magnitude smaller than that of 0.023 h^−1^ for *Denitrifying Culture I*, suggesting a much lower N_2_O reduction rate using PHB by *Denitrifying Culture II*, which was in agreement with the experimental observations about the significant high N_2_O accumulation (about 80% of nitrite was converted to N_2_O) in *Denitrifying Culture II*. The good agreement between simulations and measured results in Fig. 4 further indicated the developed model is also able to describe the N_2_O accumulation data from *Denitrifying Culture II*.

### Factors affecting PHB production and N2O accumulation

Regarding an denitrifying activated sludge system, its feeding pattern and SRT are very important, as they could regulate the length of feast and famine periods as well as the storage production^18^ and thus affect N_2_O accumulation. Further model simulations were performed to evaluate the impacts of these operational conditions on N_2_O accumulation during denitrification. Two types of widely applied activated sludge systems were investigated in terms of their steady-state denitrifying performance, namely SBR and continuous systems. Both systems were set to have the same reactor volume and loading, with influent NO_3_^−^ and Ss kept at 50 mg N/L and 300 mg-COD/L, respectively, mimicking a typical domestic wastewater condition.

**Figure 5:**
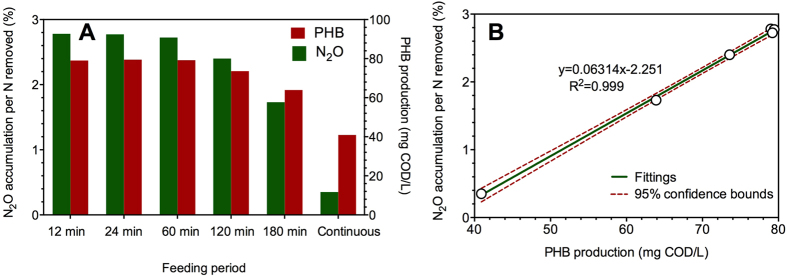
(**A**) Model simulation of the steady-state N_2_O accumulation and PHB production of a SBR performing denitrification as a function of the length of filling time. The simulation conditions are: Reactor volume = 8 L, cycle time = 6 h, SRT = 15 d, filling time = 12–180 min, decanting time = 3 min, influent loading = 16 L/d, influent Ss = 300 mg-COD/L, influent nitrate = 50 mg-N/L. In addition, extra simulation was carried out in a continuous denitrifying system with the same SRT and influent loading conditions; and (**B**) the linear relationship between the fraction of N_2_O accumulation and maximum PHB production based on the simulation results.

Firstly, the SRT was fixed at 15 d for both systems. The SBR was operated at a cycle time of 6 h, with filling time varying from 12 min to 180 min. Figure 5A shows the effect of feeding strategies on the maximum PHB production and N_2_O accumulation. With the increase of filling time from 12 min to 180 min, the fraction of N_2_O accumulation per nitrogen removed decreased from 2.8% to 1.7%. In the extreme case of the continuous system (continuous feeding), the fraction of N_2_O accumulation even dropped to 0.35%. Correspondingly, less PHB is produced during the feast period for its subsequent utilization during famine phase, i.e., from 79 mg-COD/L to 40 mg-COD/L. The relationship between the fraction of N_2_O accumulation and PHB production is positively linear at a constant SRT (Fig. 5B). The shortening of filling time with same substrate loading could provide more substrate for PHB storage process, resulting in more PHB production and shorter feast period. Therefore, more N_2_O could be accumulated during the prolonged famine period with higher PHB being available for microbial utilization.

**Figure 6:**
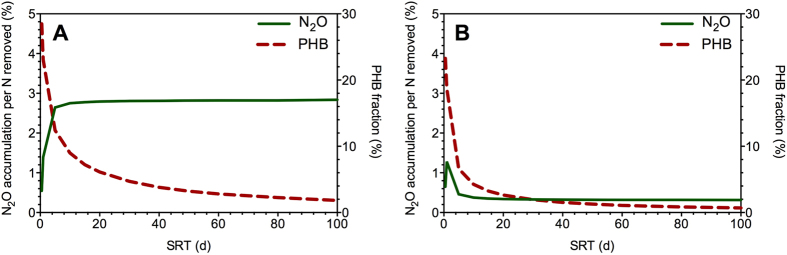
Model simulation of the effect of SRT (0.5–100 d) on N_2_O accumulation and PHB production during denitrification at steady state. (**A**) SBR system; and (**B**) continuous system. The simulation conditions for both systems are: Reactor volume = 8 L, influent loading = 16 L/d, influent Ss = 300 mg-COD/L, and influent nitrate = 50 mg-N/L. The cycle time of SBR is 6 h, consisting of a filling time of 12 min and a decanting time of 3 min.

Secondly, both systems were operated with varying SRT from 0.5 d to 100 d (Fig. 6). With the increase of SRT, the fraction of PHB produced decreased as the increase of biomass for both systems, in agreement with the observations by Ni *et al.*^19^. For the SBR, the fraction of N_2_O accumulation increased under low SRT conditions (<15 days) and then kept constant at about 2.8% from a SRT of 15 days onwards. In contrast, the fraction of N_2_O accumulation in the continuous system presented a highest value of 1.3% at a SRT of around 1 day and then gradually decreased to a relatively constant value of 0.3% due to the increasing biomass from a SRT of 20 days onwards. The difference between N_2_O accumulation trends of these two systems is due to the different pathways regulating N_2_O accumulation. For the SBR, with the increase of SRT, the substrate was consumed more quickly and thus the famine period became longer, leading to increasing N_2_O accumulation from PHB consumption pathway (famine). For the continuous system, external substrate was available continuously with lower PHB production and N_2_O accumulation was mainly contributed by external substrate consumption pathway (feast). Therefore, the overall N_2_O accumulation in the simulated denitrifying continuous system was much lower than that in the denitrifying SBR system (Fig. 6).

## Discussion

Microbial PHB storage is usually the main mechanism for the removal of readily biodegradable carbon sources in wastewater systems when operating under dynamic conditions. Microbial internal storage turnovers have been demonstrated to have a significant influence on N_2_O accumulation during heterotrophic denitrification^10^,^11^,^12^,^17^,^20^,^21^. The utilization of the produced storage polymer might be the major contributor to N_2_O accumulation during denitrification^10^,^11^,^12^. However, the previously proposed N_2_O models by heterotrophic denitrifiers are based on a four-step denitrification pathway without consideration of the role of microbial internal storage turnovers^14^,^22^. These models have been shown to be able to predict the experimental N_2_O data in denitrification when using external carbon substrate, yet not applicable to describe N_2_O accumulation by using internal storage polymers during the famine period.

In this work, a new mathematical model considering both production and consumption of internal storage products is developed to describe the N_2_O accumulation by heterotrophic denitrifiers for the first time. The validity of this developed model was confirmed by two independent data sets from different denitrifying cultures. The set of best-fit parameter values are shown in SI Table S3. The parameter values obtained were robust (R^2^ between 0.89 and 0.94) in their ability to predict nitrate, nitrite, N_2_O, and PHB dynamics under different operational conditions (feast and famine). The model evaluation results using data obtained from two different cultures indicate the developed N_2_O model is applicable for different wastewater systems. In addition, the develop model can be easily integrated with other wastewater treatment models such as the activated sludge model (ASM)-based N_2_O models for nitrification to describe overall N_2_O dynamics in wastewater treatment systems^22^,^23^,^24^.

Modeling results indicated that a much higher fraction of N_2_O could be accumulated during the famine period (2.5–5.5%, Fig. 2D) than feast period (0.4–1.3%, Fig. 2C) in denitrification due to the lower N_2_O reduction rate, based on the widely applied concept of N_2_O emission factor, i.e., N_2_O produced per N removed^11^,^22^. Correspondingly, *μ*_*4,HB,Xsto*_ (0.023 h^−1^) under the famine condition was nearly half of *μ*_*4,HB,SS*_ under the feast condition for *Denitrifying Culture I* (Table S3), in agreement with the experimental observations of lower competition capacity for electrons from internal storage polymers of nitrous oxide reductase^25^. For *Denitrifying Culture II*, the even much lower *μ*_*4,HB,Xsto*_ (0.0004 h^−1^) further confirmed this observation and coincided with the experimentally observed extremely high fraction of N_2_O accumulation (approximately 80%). The difference of the calibrated parameter values for the two independent reactor studies was likely due to the fact that the two cultures were operated in substantially different conditions (i.e., different reaction regimes, SRT, organic matters and operating strategies), resulting in distinct denitrifying microbial community in the two systems, as demonstrated by the dramatically different experimental observations in terms of PHB production and N_2_O accumulation. Additionally, the modeling results (Figs 2B and 3B) also suggested that higher nitrite accumulation could impose a more serious inhibition on nitrous oxide reductase during the famine period^26^, thus inducing more N_2_O accumulation (20–30%, Fig. 3D).

It should be noted that the developed model of this work adopted the simple “direct coupling approach”, similar as ASMN in which the carbon oxidation and nitrogen reduction processes are directly coupled in each denitrification step^14^, mainly aiming to describe and provide first insights into the role of PHB storage turnovers on N_2_O accumulation during denitrification. This approach assumes that carbon oxidation is always able to meet the electron demand by all denitrification steps, therefore discounting electron competition among these steps. However, the model of this work could be readily transformed into the recently proposed “indirect coupling approach”^15^, in which the carbon oxidation and nitrogen reduction processes are indirectly coupled through introducing electron carriers to link them for each step of denitrification, if electron competition requires to be considered. This may warrant further investigations.

The developed model and the results reported in this work are useful to select, design, and optimize biological nitrogen removal process based on denitrification in terms of N_2_O mitigation. For SBR system, increasing the filling time and decreasing the SRT would help to lower the fraction of N_2_O accumulation during denitrification by 1–2%. For the continuous system, on the contrary, keeping a relative longer SRT would decrease the N_2_O accumulation during denitrification by 0.5–1%. It should be noted that 1% increase in N_2_O emission would induce 30% increase in carbon footprint during the wastewater treatment^27^. Therefore, the information of this work would be very useful for accurate estimation and effective mitigation of N_2_O emission from wastewater treatment, especially the system operating under dynamic conditions with microbial internal storage turnovers.

In summary, a mathematical model is developed to evaluate the key role of PHB storage turnovers on N_2_O accumulation during denitrification for the first time. The developed model has been successfully applied to reproduce experimental data obtained from two different systems and under different conditions. The N_2_O emission factor (N_2_O produced per N removed) at famine phase was much higher than that at feast phase due to much less nitrogen conversion but relatively high N_2_O production at famine phase. A linear relationship between N_2_O accumulation and PHB production was revealed, suggesting a substantial effect of PHB storage on N_2_O accumulation during denitrification. The feeding pattern and SRT would alter the PHB turnovers and thus affect the N_2_O accumulation during denitrification. Increasing PHB utilization could substantially raise N_2_O accumulation due to the relatively low N_2_O reduction rate when using PHB as carbon source.

## Material and Methods

### Model development

Heterotrophic denitrifiers can produce internal storage polymers when exposed to shock loading of electron donor^16^,^22^,^28^. In this process, denitrifiers subject to consecutive periods of external carbon substrate availability (feast regime) and unavailability (famine regime), which generate an unbalanced microbial growth. Heterotrophic denitrifiers can use external carbon substrate for simultaneous growth and storage under feast conditions. After external carbon substrate is depleted, the anoxic growth on storage products takes place under famine conditions. The model developed in this work considered the four-step (from NO_3_^−^ to N_2_ via NO_2_^−^, NO and N_2_O) simultaneous anoxic storage and growth processes as well as four-step anoxic storage utilization processes for describing all potential N_2_O accumulation steps in denitrification with PHB storage turnovers. The new mathematical model that synthesizes all relevant reactions, in particular the PHB storage and utilization processes with NO_3_^−^, NO_2_^−^, NO, and N_2_O as electron acceptors, is schematically presented in Fig. 1. In the presence of high level of external carbon substrate, the uptake is driven to simultaneous growth of biomass (primary growth) and PHB storage of polymers (see Fig. 1). After external substrate exhaustion, the internal stored polymers can be used as a carbon source for a secondary growth of biomass.

The developed model describes the relationships among three biomass groups: heterotrophic denitrifiers (X_H_), storage products of denitrifiers (X_STO_), and residual inert biomass (X_I_); and six soluble compounds: 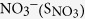, 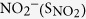, NO (S_NO_), 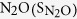, 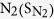, and external carbon substrate (Ss). The units are g-N m^−3^ for all nitrogenous species and g-COD m^−3^ for non-nitrogen compounds. Three groups of biological processes (see Table S1 and S2 in SI) were considered, namely, anoxic growth of denitrifiers on biodegradable substances (Process 1–4), anoxic storage of biodegradable substances by denitrifiers (Process 6–9) and anoxic growth of denitrifiers on storage products (Process 11–14), each modeled as four sequential denitrification processes from NO_3_^−^ to N_2_ via NO_2_^−^, NO and N_2_O with individual reaction-specific kinetics. An example kinetics equation of anoxic growth of denitrifiers with N_2_O and Ss (Process 4, eq. (1)), anoxic storage of Xsto with N_2_O and Ss (Process 9, eq. (2)) and anoxic growth of denitrifiers with N_2_O and Xsto (Process 14, eq. (3)) is provided as below. In addition, biomass decay (Process 5 and 10) was also included. Table S3 in the SI lists the definitions, values, units, and sources of all parameters used in the developed model.


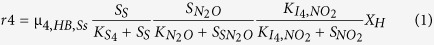



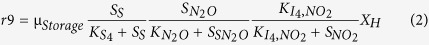



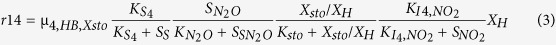


### Experimental data for model evaluation

*Denitrifying Culture I.* Experimental data from a denitrifying culture previously reported in Wu *et al.*^11^ are used for the model calibration and validation. The enriched denitrifying culture was developed in a 5.4-L lab-scale SBR fed with synthetic wastewater mainly consisting of acetate and nitrate (detailed composition described in Wu *et al.*^11^). The reactor was operated with a cycle time of 4 h consisting of 20 min filling, 150 min idle I (anoxic), 10 min idle II (aerobic, to remove N_2_ so as to improve the settlement property of the activated sludge), 40 min settling, 20 min decanting. 1.8 liters of synthetic wastewater was fed every cycle giving a hydraulic retention time (HRT) of 12 h. The SRT was kept around 10 days. This denitrifying culture reduces approximately 99% of the 100 mg-N/L nitrate contained in the feed and produces PHB as internal storage polymers, with a residue nitrite concentration below 2 mg-N/L in the effluent. More details of the reactor operation and performance can be found in Wu *et al.*^11^. Four sets of batch experiments were conducted by Wu *et al.* with this culture in a 0.5-L batch reactor at different nitrate, nitrite, acetate, and PHB levels (Table 1)^11^. Batch I and III were designed to examine the N_2_O accumulation during denitrification using external carbon substrate (i.e., acetate) for simultaneous anoxic growth and storage under feast conditions with different initial nitrite concentrations. The acetate was available during the whole periods of these experiments. Batch II and IV were designed to examine the N_2_O accumulation during denitrification using storage products (i.e., PHB) for anoxic growth under famine conditions with different initial nitrite concentrations, which were initiated after accumulation step, centrifuged and re-suspended of sludge in a solution of the synthetic wastewater but without the addition of external carbon source. In each batch experiment, initial nitrate concentration was set at around 100 mg-N/L. Mixed liquor and gas samples were taken periodically for NO_3_^−^, NO_2_^−^, acetate, PHB, and N_2_O analysis, respectively. More detailed experimental setup and analysis methods can be found in Wu *et al.*^11^.

*Denitrifying Culture II.* A nitrite-reduction denitrifying culture was enriched in an SBR with a working volume of 4 L for six months^17^. The reactor was operated with a cycle time of 48 h: i) anaerobic phase (24 h) in which acetate was added and assimilated as PHB during a fast fill (0.5 h) followed by a react period (23.5 h); ii) anoxic phase (23.5 h), in which a nitrite-rich effluent from a partial nitrifying reactor was added for oxidation of PHB coupled to reduction of NO_2_^−^ to N_2_O during a fast fill (0.1 h) followed by a react period (23.4 h); iii) settling phase (25 min); and iv) decant phase (5 min). In each cycle, 100 mL of stock sodium acetate solution (122 mM) was added to initiate the anaerobic phase for PHB production, resulting in an initial acetate concentration of 250 mg-COD/L. After completion of the anaerobic phase, 180 mL of liquid from the partial nitrifying reactor (∼1,400 mg-N/L NO_2_^−^) was added to initiate the anoxic phase for PHB utilization, leading to an initial nitrite concentration of 85 mg-N/L. This reactor achieved a 96% nitrogen removal, with 78% nitrogen from nitrite being converted to N_2_O. The cycle profile of this nitrite-reduction culture was used for model evaluation in terms of N_2_O accumulation when using storage products as carbon source for anoxic growth under famine conditions. The sampling and analysis methods were similar to those for Culture I except that N_2_O was measured using an online sensor.

### Model calibration and validation

The developed model includes 28 stoichiometric and kinetic parameters as summarized in Table S3 in SI. About 21 of these model parameter values are well established in previous studies. Thus, literature values were directly adopted for these parameters (SI Table S3). The NO reduction related parameters is beyond the ability of measurement since NO was not added in any tests given its toxicity to bacteria. Indeed, NO reduction is usually prioritized by bacteria to avoid its toxicity, thus a relatively high value of *μ*_*3,HB,SS*_ and a low value of *K*_*NO*_ from literature are used in this model (Table S3) to ensure there is no accumulation of NO. The remaining seven parameters, i.e., anoxic growth rate on nitrate and Ss (*μ*_*1,HB,SS*_), anoxic growth rate on nitrite and Ss (*μ*_*2,HB,SS*_), anoxic growth rate for on N_2_O and Ss (*μ*_*4,HB,SS*_), anoxic storage rate of Ss (*μ*_*storage*_), anoxic growth rate on nitrate and X_STO_ (*μ*_*1,HB,Xsto*_), anoxic growth rate on nitrite and X_STO_ (*μ*_*2,HB,Xsto*_), and anoxic growth rate on N_2_O and X_STO_ (*μ*_*4,HB,Xsto*_), which are unique to the proposed model and the key parameters governing the N_2_O accumulation, are then calibrated using experimental data (SI Table S3). Specifically, the simultaneous growth and storage related parameters (*μ*_*1,HB,SS*_, *μ*_*2,HB,SS*_, *μ*_*4,HB,SS*_ and *μ*_*storage*_) are calibrated using data from feast phase test (external carbon consumption). The storage utilization related parameters (*μ*_*1,HB,Xsto*_, *μ*_*2,HB,Xsto*_ and *μ*_*4,HB,Xsto*_) are calibrated using data from famine phase test (storage consumption).

Experimental data (nitrate, nitrite, N_2_O, PHB) from Batch I (feast) and II (famine) of *Denitrifying Culture I* with different electron donors (acetate or PHB) in the absence of initial nitrite were used to calibrate the model. The parameter values were estimated by minimizing the sum of squares of the deviations between the measured data and the model predictions using the secant method embedded in AQUASIM 2.1d^29^,^30^,^31^,^32^. Model validation was then carried out with the calibrated model parameters using the other two sets of experimental data (nitrate, nitrite, N_2_O, PHB) from Batch III (feast) and IV (famine) of *Denitrifying Culture I* in the presence of initial nitrite (different conditions from the calibration tests, as shown in Table 1).

To further verify the validity and applicability of the model, we also applied the model to evaluate the NO_2_^−^, N_2_O and PHB data from *Denitrifying Culture II*, with particular focus on N_2_O accumulation from PHB utilization processes as this culture showed extremely high N_2_O accumulation when using storage polymers as electron donor for nitrite reduction. To this end, two model parameters (*μ*_*2,HB,Xsto*_ and *μ*_*4,HB,Xsto*_) were calibrated for *Denitrifying Culture II* using the cycle study data with PHB consumption under the famine condition.

## Additional Information

**How to cite this article**: Liu, Y. *et al.* Evaluating the Role of Microbial Internal Storage Turnover on Nitrous Oxide Accumulation During Denitrification. *Sci. Rep.* **5**, 15138; doi: 10.1038/srep15138 (2015).

## Supplementary Material

Supplementary Information

## Figures and Tables

**Table 1:** The initial batch experiment conditions using denitrifying culture I according to Wu *et al.* 11.

Experiment	Nitrate (mg-N/L)	Nitrite (mg-N/L)	Acetate (mg-COD/L)	PHB (mg-COD/L)
Batch I	100	0	803	0
Batch II	100	0	0	165
Batch III	100	20	803	0
Batch IV	100	20	0	165
