# Supplementary material for: Evaluating the Role of Microbial Internal Storage Turnover on Nitrous Oxide Accumulation During Denitrification
Source: Sci Rep. 2015 Oct 14;5:15138. doi: 10.1038/srep15138 (PMC4604521; doi:10.1038/srep15138)
Supplement: Supplementary Information [file srep15138-s1.pdf]

Supporting Information

Evaluating the Role of Microbial Internal Storage Turnover on Nitrous Oxide Accumulation during Denitrification

Yiwen Liu, Lai Peng, Jianhua Guo, Xueming Chen, Zhiguo Yuan, Bing-Jie Ni*

Advanced Water Management Centre, The University of Queensland, St. Lucia, Brisbane, QLD 4072, Australia

***Corresponding author:**

Bing-Jie Ni, P: +61 7 3346 3230; F: +61 7 3365 4726; E-mail: b.ni@uq.edu.au

The following is included as supporting information for this paper:

number of pages: 4

number of tables: 3

number of figures: 0

Table S1. Process Kinetic Rate Equations for the Developed Model

Process	Kinetics rates expressions
Anoxic growth of Heterotrophic Bacteria (HB) on external carbon substrate (Ss)	
1. Anoxic growth of HB with nitrate and Ss	$\mu_{1,HB,Ss}\frac{S_S}{K_{S1}+S_S}\frac{S_{NO3}}{K_{NO3}+S_{SNO3}}\frac{K_{I1,NO2}}{K_{I1,NO2}+S_{NO2}}X_H$
2. Anoxic growth of HB with nitrite and Ss	$\mu_{2,HB,Ss}\frac{S_S}{K_{S2}+S_S}\frac{S_{NO2}}{K_{NO2}+S_{SNO2}}\frac{K_{I2,NO2}}{K_{I2,NO2}+S_{NO2}}X_H$
3. Anoxic growth of HB with NO and Ss	$\mu_{3,HB,Ss}\frac{S_S}{K_{S3}+S_S}\frac{S_{NO}}{K_{NO}+S_{SNO}}\frac{K_{I3,NO2}}{K_{I3,NO2}+S_{NO2}}X_H$
4. Anoxic growth of HB with N_2O and Ss	$\mu_{4,HB,Ss}\frac{S_S}{K_{S4}+S_S}\frac{S_{N2O}}{K_{N2O}+S_{SN2O}}\frac{K_{I4,NO2}}{K_{I4,NO2}+S_{NO2}}X_H$
5. Decay of HB	$b_H X_H$
Anoxic storage of Ss for X_{sto} production by HB	
6. Anoxic storage of Xsto with nitrate and Ss	$\mu_{Storage}\frac{S_S}{K_{S1}+S_S}\frac{S_{NO3}}{K_{NO3}+S_{SNO3}}\frac{K_{I1,NO2}}{K_{I1,NO2}+S_{NO2}}X_H$
7. Anoxic storage of Xsto with nitrite and Ss	$\mu_{Storage}\frac{S_S}{K_{S2}+S_S}\frac{S_{NO2}}{K_{NO2}+S_{SNO2}}\frac{K_{I2,NO2}}{K_{I2,NO2}+S_{NO2}}X_H$
8. Anoxic storage of Xsto with NO and Ss	$\mu_{Storage}\frac{S_S}{K_{S3}+S_S}\frac{S_{NO}}{K_{NO}+S_{SNO}}\frac{K_{I3,NO2}}{K_{I3,NO2}+S_{NO2}}X_H$
9. Anoxic storage of Xsto with N_2O and Ss	$\mu_{Storage}\frac{S_S}{K_{S4}+S_S}\frac{S_{N2O}}{K_{N2O}+S_{SN2O}}\frac{K_{I4,NO2}}{K_{I4,NO2}+S_{NO2}}X_H$
10. Decay of Xsto	$b_{sto}X_{sto}$
Anoxic growth of HB on Xsto	
11. Anoxic growth of HB with nitrate and Xsto	$\mu_{1,HB,Xsto}\frac{K_{S1}}{K_{S1}+S_S}\frac{S_{NO3}}{K_{NO3}+S_{SNO3}}\frac{X_{sto}/X_H}{K_{sto}+X_{sto}/X_H}\frac{K_{I1,NO2}}{K_{I1,NO2}+S_{NO2}}X_H$
12. Anoxic growth of HB with nitrite and Xsto	$\mu_{2,HB,Xsto}\frac{K_{S2}}{K_{S2}+S_S}\frac{S_{NO2}}{K_{NO2}+S_{SNO2}}\frac{X_{sto}/X_H}{K_{sto}+X_{sto}/X_H}\frac{K_{I2,NO2}}{K_{I2,NO2}+S_{NO2}}X_H$
13. Anoxic growth of HB with NO and Xsto	$\mu_{3,HB,Xsto}\frac{K_{S3}}{K_{S3}+S_S}\frac{S_{NO}}{K_{NO}+S_{SNO}}\frac{X_{sto}/X_H}{K_{sto}+X_{sto}/X_H}\frac{K_{I3,NO2}}{K_{I3,NO2}+S_{NO2}}X_H$
14. Anoxic growth of HB with N_2O and Xsto	$\mu_{4,HB,Xsto}\frac{K_{S4}}{K_{S4}+S_S}\frac{S_{N2O}}{K_{N2O}+S_{SN2O}}\frac{X_{sto}/X_H}{K_{sto}+X_{sto}/X_H}\frac{K_{I4,NO2}}{K_{I4,NO2}+S_{NO2}}X_H$

Table S2. Stoichiometric Matrix for the Developed Model

Variable<br>Process	S_{NO3}<br>N	S_{NO2}<br>N	S_{NO}<br>N	S_{N2O}<br>N	S_{N2}<br>N	Ss<br>COD	X_H<br>COD	X_I<br>COD	X_{sto}<br>COD
1	$-\frac{1-Y_H}{1.14Y_H}$	$\frac{1-Y_H}{1.14Y_H}$				$-\frac{1}{Y_H}$	1		
2		$-\frac{1-Y_H}{0.57Y_H}$	$\frac{1-Y_H}{0.57Y_H}$			$-\frac{1}{Y_H}$	1		
3			$-\frac{1-Y_H}{0.57Y_H}$	$\frac{1-Y_H}{0.57Y_H}$		$-\frac{1}{Y_H}$	1		
4				$-\frac{1-Y_H}{0.57Y_H}$	$\frac{1-Y_H}{0.57Y_H}$	$-\frac{1}{Y_H}$	1		
5							-1	f_I	
6	$-\frac{1-Y_{storage}}{1.14Y_{storage}}$	$\frac{1-Y_{storage}}{1.14Y_{storage}}$				$-\frac{1}{Y_{storage}}$			1
7		$-\frac{1-Y_{storage}}{0.57Y_{storage}}$	$\frac{1-Y_{storage}}{0.57Y_{storage}}$			$-\frac{1}{Y_{storage}}$			1
8			$-\frac{1-Y_{storage}}{0.57Y_{storage}}$	$\frac{1-Y_{storage}}{0.57Y_{storage}}$		$-\frac{1}{Y_{storage}}$			1
9				$-\frac{1-Y_{storage}}{0.57Y_{storage}}$	$\frac{1-Y_{storage}}{0.57Y_{storage}}$	$-\frac{1}{Y_{storage}}$			1
10									-1
11	$-\frac{1-Y_{H,sto}}{1.14Y_{H,sto}}$	$\frac{1-Y_{H,sto}}{1.14Y_{H,sto}}$					1		$-\frac{1}{Y_{H,sto}}$
12		$-\frac{1-Y_{H,sto}}{0.57Y_{H,sto}}$	$\frac{1-Y_{H,sto}}{0.57Y_{H,sto}}$				1		$-\frac{1}{Y_{H,sto}}$
13			$-\frac{1-Y_{H,sto}}{0.57Y_{H,sto}}$	$\frac{1-Y_{H,sto}}{0.57Y_{H,sto}}$			1		$-\frac{1}{Y_{H,sto}}$
14				$-\frac{1-Y_{H,sto}}{0.57Y_{H,sto}}$	$\frac{1-Y_{H,sto}}{0.57Y_{H,sto}}$		1		$-\frac{1}{Y_{H,sto}}$

Table S3. Kinetic and Stoichiometric Parameters of the Developed Model

Parameter	Definition	Values	Unit	Source
Stoichiometric parameters				
Y_H	yield coefficient for growth on Ss	0.4	g COD g^{-1} COD	(1)
$Y_{storage}$	yield coefficient for storage on Ss	0.55	g COD g^{-1} N	(1)
$Y_{H,sto}$	yield coefficient for growth on Xsto	0.67	g COD g^{-1} COD	(1)
f_I	Fraction of residual inert biomass (X_I)	0.1	g COD g^{-1} COD	(2)
Kinetic parameters				
$\mu_{1,HB,SS}$	maximum anoxic growth rate on nitrate and Ss	0.189	h^{-1}	(3)
$\mu_{2,HB,SS}$	maximum anoxic growth rate on nitrite and Ss	0.036	h^{-1}	(3)
$\mu_{3,HB,SS}$	maximum anoxic growth rate on NO and Ss	0.142	h^{-1}	(4)
$\mu_{4,HB,SS}$	maximum anoxic growth rate on N_2O and Ss	0.043	h^{-1}	(3)
b_H	decay rate coefficient of HB	0.026	h^{-1}	(4)
K_{S1}	half saturation coefficients of S_S for nitrate reduction	5	g COD m^{-3}	(4)
K_{S2}	half saturation coefficients of S_S for nitrite reduction	20	g COD m^{-3}	(2)
K_{S3}	half saturation coefficients of S_S for NO reduction	2.4	g COD m^{-3}	(4)
K_{S4}	half saturation coefficients of S_S for N_2O reduction	20	g COD m^{-3}	(2)
K_{NO3}	half saturation coefficients of S_{NO3}	0.251	g N m^{-3}	(4)
K_{NO2}	half saturation coefficients of S_{NO2}	0.81	g N m^{-3}	(4)
K_{NO}	half saturation coefficients of S_{NO}	0.0021	g N m^{-3}	(4)
K_{N2O}	half saturation coefficients of S_{N2O}	0.0052	g N m^{-3}	(4)
$K_{I1,NO2}$	NO_2^- inhibition constant for nitrate reduction	12	g N m^{-3}	(5)
$K_{I2,NO2}$	NO_2^- inhibition constant for nitrite reduction	10	g N m^{-3}	(5)
$K_{I3,NO2}$	NO_2^- inhibition constant for NO reduction	17	g N m^{-3}	(5)

$K_{I4,NO2}$	NO_2^- inhibition constant for N_2O reduction	8	g N m^{-3}	(5)
$\mu_{storage}$	maximum storage rate	0.03	h^{-1}	(3)
b_{sto}	decay rate coefficient of Xsto	0.004	h^{-1}	(1)
$\mu_{1,HB,Xsto}$	maximum anoxic growth rate on nitrate and Xsto	0.033	h^{-1}	(3)
$\mu_{2,HB,Xsto}$	maximum anoxic growth rate on nitrite and Xsto	0.024	h^{-1}	(3)
$\mu_{3,HB,Xsto}$	maximum anoxic growth rate on NO and Xsto	0.142	h^{-1}	(4)
$\mu_{4,HB,Xsto}$	maximum anoxic growth rate on N_2O and Xsto	0.023	h^{-1}	(3)
K_{sto}	half saturation coefficients of Xsto	1	g COD m^{-3}	(1)

Source: (1) Ni et al., 2008; (2) Hiatt et al., 2008; (3) Estimated in this study using experimental data from *Denitrifying Culture I*; (4) Ni et al., 2011; (5) von Schulthess et al., 1996.
